# Porcine Torovirus (PToV)—A Brief Review of Etiology, Diagnostic Assays and Current Epidemiology

**DOI:** 10.3389/fvets.2019.00120

**Published:** 2019-04-18

**Authors:** Zhang-Min Hu, Yong-Le Yang, Ling-Dong Xu, Bin Wang, Pan Qin, Yao-Wei Huang

**Affiliations:** Key Laboratory of Animal Virology of Ministry of Agriculture and Institute of Preventive Veterinary Medicine, College of Animal Sciences, Zhejiang University, Hangzhou, China

**Keywords:** porcine torovirus, etiology, epidemiology, diagnostic assays, recombination

## Abstract

Porcine torovirus (PToV) is a potential enteric swine pathogen, found at especially high rates in piglets with diarrhea. It was first reported in the Netherlands in 1998 and has emerged in many countries around the world. Infections are generally asymptomatic and have not directly caused large economic losses, though co-infections with other swine pathogens and intertype recombination may lead to unpredictable outcomes. This review introduces progress in PToV research regarding its discovery, relationship with other Toroviruses, virion morphological characteristics, genetic structure and variation, recent epidemiology, diagnostic methods, and possibilities for future research.

## History

Toroviruses (ToV) (order *Nidovirales*; suborder *Tornidovirineae*; family *Tobaniviridae*; subfamily *Torovirinae*; genus *Torovirus;* subgenus *Renitovirus)* are responsible for infections leading to gastroenteritis in animals and humans (1–5). There are three recognized species in the *Renitovirus* subgenus: porcine torovirus (PToV); bovine torovirus (BToV), and equine torovirus (EToV) (Virus Taxonomy: 2018b Release (MSL #34); https://talk.ictvonline.org/taxonomy/). Human torovirus (HToV) is used to be the member of the *Torovirus* genus, according to the 2017 Release of Virus Taxonomy (MSL #32). Neutralizing activity against EToV has been found in the sera of other animals (cattle, goats, sheep, swine, rabbits, mice), providing serological evidence for the existence of ToVs in other animals (5).

EToV (also called Berne virus [BEV]) was the first to be discovered, and is the prototype species of the genus *Torovirus*. EToV was initially isolated in 1972 (but not reported until 1983) from a rectal swab taken from a horse in Berne, Switzerland which showed pseudomembranous enteritis, miliary granulomas and necrosis in the liver at necropsy. The isolated pathogenic agent couldn't be neutralized by antibodies against equine viruses known at the time. By electron microscopy, the virions appeared pleomorphic, mostly with smooth surfaces and spherical, though some were C-shaped, and some particles in damaged membranes had a “sausage-like” internal structure with transverse striations (1).

Another unclassified virus, BToV (also called Breda virus [BRV]) was discovered in 1982 in calves with diarrhea in Breda, Iowa, and confirmed to have antigenic differences from known diarrhea-related bovine viruses. The isolate was infectious when orally inoculated into gnotobiotic and conventionally reared calves (2).

In 1984, a similar virus was found in the feces of diarrheic patients (mainly children under 5 years-old) in England and the United States, with virions about 100 nm in diameter and a 7–9 nm capsule on the edge (3).

Previously, ToV-like agents had been detected in swine in many countries by electron microscopy or neutralization assays performed with EToV antibodies (5–8). In 1998, PToV was first detected and characterized in fecal samples from piglets in the Netherlands (4). Immunoelectron microscopy showed the elongated, 120- by 55-nm particles in fresh material. By sequence analysis, the N protein gene of PToV only has 68% sequence identity with BToV and EToV, which share 88% between them (4). PToV has since been reported in Canada, the United States, South Africa, China, Korea, and many European countries, such as the United Kingdom, Italy, Belgium, Hungary, and Spain (5–14).

## Etiology

### PToV Structure

ToVs are enveloped viruses with positive-sense single-stranded RNA genomes. PToV particles appear elongated in fresh samples, about 120 nm long and 55 nm wide, as observed by EM. After repeated freezing and thawing, virions have multiple forms, including round, kidney- and torus-shaped particles (4). The nucleocapsid is formed by N protein and viral RNA, surrounded by an envelope that contains the triple-spanning membrane (M) protein (10). On the surface of particles, two kinds of projections have been identified: longer protrusions (about 19 nm) with a drumstick or petal shape, considered to be spike (S) proteins; and shorter ones (6 nm in length) speculated to be the hemagglutinin-esterase (HE) (4, 15).

### PToV Genome

**Figure 1:**
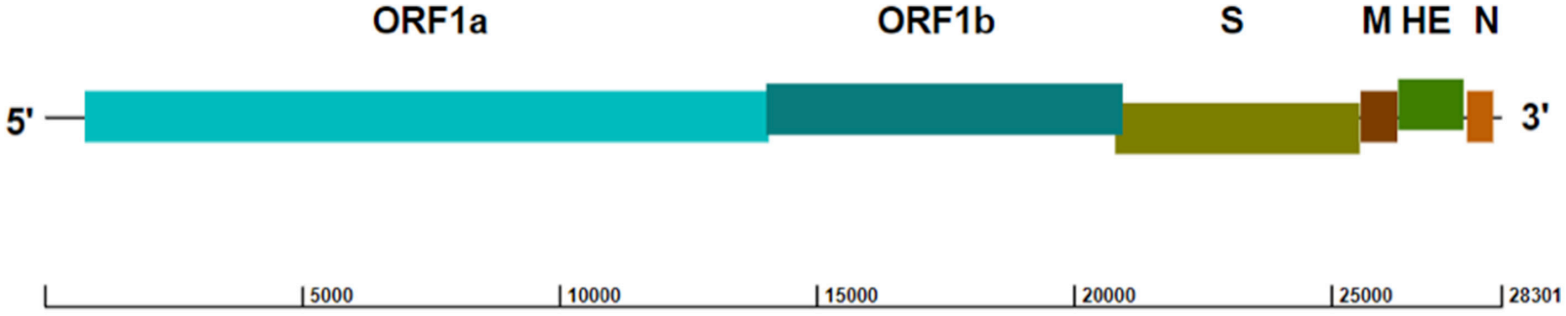
Genome structure of PToV strain SH1 (GenBank accession no. JQ860350). The ORFs encoding the replicase polyproteins (ORF1a and ORF1b) and structural proteins spike (S), membrane (M), hemagglutinin-esterase (HE), and nucleocapsid (N) are indicated.

ToV genomes are nonsegmented, polyadenylated, and 25–30 kb in size, with similar organization to other coronaviruses (16, 17). Both the 5′ and 3′ termini of the genome have a non-translated region (NTR), and the 5′ two-thirds of the ToV genome contains two large, overlapping open reading frames (ORF1a [13,254 bp] and ORF1b [6,875 bp]) connected by a frameshift (Figure 1), which encode two replicase polyproteins. There are four smaller ORFs downstream encoding the structural proteins (S [4,722 bp]; M [702 bp]; HE [1,284 bp]; and N [492 bp]), which are expressed through a 3′-coterminal nested set of subgenomic mRNAs (17–21). The first genomic sequence of PToV from Shanghai, China (strain SH1; GenBank accession no. JQ860350) was 28,301 bp in length, and had 79% sequence identity with BToV (21).

### PToV Structural Proteins

The mature ToV S protein contains two functional domains, S1 and S2, which mediate receptor specificity and determine viral tropism; S1 can bind independently to cellular receptors whereas S2 mediates fusion of the viral and cell membranes (22). Based on their electrophoretic mobility upon endoglycosidase F treatment, it has been demonstrated that the putative cleavage site, composed of five consecutive arginine residues, plays a role in post-translational processing *in vivo*. There are structural features in common with other coronavirus S proteins, such as the N-terminal signal sequence, an assumed C-terminal transmembrane anchoring domain, two heptad-repeat domains and a probable “trypsin-like” cleavage site (23). Due to its important role in host receptor binding, the S protein is the major target of neutralizing antibody responses.

The ToV M protein, previously referred to as the envelope protein, has three transmembrane α-helices in its N-terminal part. Its C-terminal part is exposed on the cytoplasmic face of the membrane, a feature of coronavirus M proteins. This protein lacks a cleavage signal sequence, so it is suspected that one of the hydrophobic transmembrane domains acts as an internal signal sequence (24).

Observed by EM, the HE protein is distributed on the virion surface as small projections. It is a class I membrane protein, and a member of the receptor destroying enzyme (RDE) protein family. There are two main domains identified in the monomer: the enzymatic acetyl-esterase region (E); and the receptor binding or lectin domain (25). The HE protein specifically but reversibly binds to mucopolysaccharides, mediating adhesion of virions to the intestinal wall. Through binding to 9-O-acetylated receptors then cleaving and rebinding to glycosylated surfaces (26, 27), virions can theoretically migrate through the mucus layer, thus promoting infection (18).

The open torus-shaped core of ToV is formed by the viral genome binding to the phosphorylated N protein, which is the only viral RNA binding protein. This 18.7 kDa protein is the most abundant protein among the ToV virions (28). Structural predictions of N protein have revealed the presence of four *N*-glycosylation sites [associated with antigenicity and immunogenicity (29)], two protein kinase C phosphorylation sites, one casein kinase II phosphorylation site, and an N-myristoylation site.

### PToV Strain Variability

Sequencing studies around the world have shown significant variation among PToV isolates by geographic region. The genetic diversity of PToV in three Korean farms was studied in 2010, showing low nucleotide and deduced amino acid identities of partial S genes. Among the Korean PToV strains there was 73.5–94.1 and 71.4–95.0% identity in nucleotide and deduced amino acid sequences, respectively, whereas comparison with the Netherland strain Markelo revealed 74.0–93.1% and 73.2–95.5% identity, respectively. In addition, phylogenetic analysis of the N gene showed that different PToV strains were emerging in Korea, and even within the same farm (11).

The complete genome of the first PToV strain identified in the United States (PToV-NPL/2013, GenBank accession no. KM403390) contained 28,305 bp, with 92% identity to PToV-SH1. The predicted S, M, HE, and N proteins shared 94, 99, 92, and 96% amino acid identity with PToV-SH1, respectively. The gene encoding HE had 80–95% identity to other PToV strain sequences in GenBank, while the N and M genes were 95 and 93–97%, respectively, indicating that HE is more diverse among PToV strains (14).

Through phylogenetic analysis, the M gene sequences of 19 novel PToV strains from Sichuan, China and 21 ToV strains in GenBank could be classified into two genotypes (I & II). All of the novel Sichuan strains belonged to genotype I along with two Korean sequences (GU-07-56-11 and GU-07-56-22), whereas all other representative Korean, Netherlands and Canadian strains from GenBank belonged to genotype II. Putative amino acid sequence identities of the M gene were 99.1–99.6% among the 19 Sichuan strains and 97.4–99.6% between Sichuan and foreign strains, demonstrating high conservation of the M gene (30). Since the M gene sequences available were highly conserved, genotyping of PToV based on the M gene may be meaningless.

The sequence differences between the four *Torovirus* species range from 30 to 40%, which makes it easy to distinguish between them. However, evidence of intertype recombination was found in 2003, where BToV variants had emerged from recombination with PToV at the 3′ end of the HE/N genes and the 3′-NTR (31). Similar recombination events have also been found in ORF1a, ORF1b, and the 5′-NTR (32). Furthermore, some PToV and BToV variants have been isolated with chimeric HE genes, speculatively from recombination with some unknown ToVs (31). This ability for recombination underlines the potential for cross-species transmission and even the risk of zoonosis.

### Inter-order Recombination Between ToV and Enterovirus in Swine

Interestingly, evidences of inter-order recombination between ToV (order *Nidovirales*) and the order of *Picornavirales* were discovered in different geographical locations most recently. The porcine enterovirus G (EV-G) genome with an insertion of ToV papain-like cysteine protease gene (PLCP) at the 2C/3A junction was detected in the feces of diarrheal pigs from farms in the United States, Belgium, Japan, China, and South Korea (33–38). The inserted PLCP sequences had lengths varied in strains from different geographical locations, ranging from 194 to 223 amino acids, which form a separate cluster distantly related to those of PToV, BToV, and EToV, suggesting that these viruses might have a common ancester. Since a mutant virus without the insertion by reverse genetics produced impaired growth and higher expression levels of innate immune genes in infected cells (36), the PLCP sequence might help the EV-G-PLCP strains establish a new host immune evasion strategy and, in some cases, determine its pathogenic potential.

## Diagnostic Assays

Immunoelectron microscopy was used to detect PToV in the early years. However, this method was costly, time-consuming, and not suitable for processing a large number of samples. In addition, the polymorphism of ToV particles (4) decreases the specificity and accuracy of this detection method, and the fact that viral particles are not shed in the feces for a long duration decreases the likelihood of detection.

Neutralization assays using EToV virions obtained from cell culture (39) and an indirect ELISA method using BToV virions obtained from infected gnotobiotic calves (40) have been applied to serological diagnosis of PToV by detecting cross-reacting antibodies. However, the difficulty of virion purification and lost sensitivity resulting from use of heterologous antigens make these assays inappropriate for widespread use. The lack of a PToV culture system and infection model makes it difficult to obtain a large number of virions, hampering development of diagnostic methods and epidemiology studies. The only method available for serological diagnosis of PToV is an indirect ELISA based on a recombinant His-tagged N protein expressed in a baculovirus system. Serum samples (*n* = 15) collected from 6 to 8 week-old healthy piglets from a farm located in Galicia, Spain, tested by this method obtained a positive rate of 100%, whereas the positive rates when tested by western blot and neutralization assay of EToV virions were 60% (9/15) and 93% (14/15), respectively (10). It could be seen that this ELISA method had a high sensitivity, but its specificity may be slightly worse. The indirect ELISA based on PToV-HE protein is less sensitive and may have false negative results because of the diversity of HE proteins and the antigenic differences between HE lineages (14, 25). Development of other ELISA diagnostic methods based upon the PToV S protein is needed, according to experiences from porcine coronavirus such as porcine epidemic diarrhea virus (PEDV) (41).

Currently, PCR is the most widely used diagnostic assay, with the advantages of being low-cost, convenient, and highly sensitive and specific. Apart from conventional reverse transcription PCR (RT-PCR) (4, 10, 13, 31), quantitative RT-PCR (qRT-PCR) methods with primer pairs derived from the N gene (9, 39) have also been established to detect PToV and investigate epidemiology, since the N protein is the most abundant protein in the virus and has a high sequence conservation. A one-step SYBR Green qRT-PCR based on the PToV M gene was shown to be more sensitive than conventional RT-PCR and nested PCR (42). A multiplex RT-PCR method was developed for simultaneous detection of porcine kobuvirus (PKV), porcine astrovirus (PAstV) and PToV using primers based on their M gene sequences (43). Furthermore, an RT-LAMP method based on 4 specific primers from the N gene was developed for the quick detection of PToV (44).

## Epidemiology

The worldwide distribution of PToV has been proven, with a high infection rate in pigs. However, due to limitations of diagnostic assays and asymptomatic infections (4, 13), there are not many reports on epidemiology. In 1998, Kroneman et al. performed a neutralization assay using EToV to detect cross-reacting antibodies and found that 81.4% (96/118) of the pig serum samples collected from farms in the Netherlands contained EToV-neutralizing antibodies (4). A qRT-PCR method was applied to detect PToV in rectal swabs collected from piglets at a farm in northeastern Spain in 2010, with a positive detection rate of 39.6% (19/48) (9). A longitudinal serological and virological study of PToV in Spain detected serum antibody levels by N protein ELISA, and fecal shedding by qRT-PCR based on the N gene. Seroprevalence in one hundred and twenty piglets at 1, 3, 7, 11, and 15 weeks-of-age was 92, 58, 91, 100, and 100% positive, respectively, and the corresponding 30 sows were all seropositive, reflecting the process of maternal antibody decline and subsequent immune response. As for fecal shedding in a 36-piglet subpopulation, 92% (33/36) of piglets had detectable PToV RNA at some age (39). Another epidemiological study in Spanish farms was done in 2012, with serum samples collected from 100 farms tested by N protein ELISA, revealing a total seroprevalence of 95.7% (2550/2664) and prevalence at different ages ranging from 59.4 to 99.6%. The lowest seroprevalence was detected in 3-week-old piglets (98/165) (45).

Shin et al. examined the prevalence of PToV in Korea in 2007, revealing 6.4% (19/295) of diarrheic pig samples were positive by RT-PCR (11). Among samples from diarrheic pigs collected in Korea during 2004–2005 and 2007, 36% (31/86) were positive by SYBR Green qRT-PCR (42). RT-PCR targeting the S gene was used to test stool and intestinal samples of diarrheic piglets from 20 farms in southwest China collected in the winter of 2011, with 45% (9/20) farms positive for PToV. In addition, 7 of those 9 farms had mixed infection with other swine viruses including PEDV, PKV, porcine rotavirus group A (PRV-A), transmissible gastroenteritis virus (TGEV), PAstV and mammalian orthoreovirus (MRV) (12). In Sichuan Province in the southwest of China, 872 fecal samples collected from diarrheic swine in 2011–2013 were tested by RT-PCR based on the conserved region of the S gene. An overall positive rate of 37.96% (331/872) was found, with positive co-infection with PEDV, TGEV or PRV-A in 4.1% (36/872) of these samples. Among the different ages tested, piglets at 1–3 weeks-of-age had the highest infection rate of 42.47% (295/697) (30).

## Discussion

Most of our existing knowledge about ToV is based on the study of BToV and EToV, or the members of the *Coronaviridae*. The lack of an adaptive culture system and infection model to grow the virus hampers the study of viral characteristics and development of diagnostic tools. On the other hand, as PToV has not caused great economic losses, people do not pay attention to it, resulting in a lack of research on treatment and prevention.

The limited studies of sequence diversity may impede development of accurate diagnostic assays and vaccine production. Especially, a lack of study of the variability of the S gene also limits our understanding of the serology of PToV. So far, though many test methods have been established, there are no commercially available diagnostic kits. The expression of structural proteins by various means is important in order to screen for antibodies against PToV, and monoclonal antibodies are needed for further research on important topics like the mechanism of pathogenesis. In particular, the sequence of structural genes, as well as their processing and modification, may affect host specificity of the virus.

Intertype recombination events that have occurred in Europe (31) and Japan (32), among other places, remind us not to underestimate the danger posed by PToV from the possibility of cross-species infection. The mechanism of pathogenesis of PToV is still unclear, and its role during co-infections with other swine enteric pathogens such as PRV A, PAstV, PEDV, TGEV, PKV and *Salmonella* spp. is unknown (11, 30, 46). Considering the prevalence of asymptomatic PToV infections, more research is needed to explore whether it may aggravate the diseases caused by other swine pathogens.

## Author Contributions

Z-MH, Y-WH wrote the manuscript. All other co-authors edited the manuscript.

### Conflict of Interest Statement

The authors declare that the research was conducted in the absence of any commercial or financial relationships that could be construed as a potential conflict of interest.
